# Surveillance of testicular microlithiasis?: Results of an UK based national questionnaire survey

**DOI:** 10.1186/1471-2490-6-8

**Published:** 2006-03-15

**Authors:** Subramanian Ravichandran, Richard Smith, Philip A Cornford, Mark VP Fordham

**Affiliations:** 1 Specialist Registrar in Urology, University Hospital Aintree, Liverpool, L9 7AL, UK; 2 SHO in Urology, Royal Liverpool University Hospital, Prescot Road, Liverpool, UK; 3 Consultant Urologist, Royal Liverpool University Hospital, Prescot Road, Liverpool, UK; 4 17, Milfield, Neston, Cheshire, CH64 3TF, UK

## Abstract

**Background:**

The association of testicular microlithiasis with testicular tumour and the need for follow-up remain largely unclear.

**Methods:**

We conducted a national questionnaire survey involving consultant BAUS members (BAUS is the official national organisation (like the AUA in USA) of the practising urologists in the UK and Ireland), to provide a snapshot of current attitudes towards investigation and surveillance of patients with testicular microlithiasis.

**Results:**

Of the 464 questionnaires sent to the BAUS membership, 263(57%) were returned. 251 returns (12 were incomplete) were analysed, of whom 173(69%) do and 78(31%) do not follow-up testicular microlithiasis. Of the 173 who do follow-up, 119(69%) follow-up all patients while 54(31%) follow-up only a selected group of patients. 172 of 173 use ultra sound scan while 27(16%) check tumour makers. 10(6%) arrange ultrasound scan every six months, 151(88%) annually while 10(6%) at longer intervals. 66(38%) intend to follow-up these patients for life while, 80(47%) until 55 years of age and 26(15%) for up to 5 years. 173(68.9%) believe testicular microlithiasis is associated with CIS in < 1%, 53(21%) think it is between 1&10% while 7(3%) believe it is > 10%. 109(43%) believe those patients who develop a tumour, will have survival benefit with follow-up while 142(57%) do not. Interestingly, 66(38%) who follow-up these patients do not think there is a survival benefit.

**Conclusion:**

There is significant variability in how patients with testicular microlithiasis are followed-up. However a majority of consultant urologists nationally, believe surveillance of this patient group confers no survival benefit. There is a clear need to clarify this issue in order to recommend a coherent surveillance policy.

## Background

Ever since Doherty et al [1] described testicular microlithiasis on ultrasound scan in the late 80 s, the interest in this subject had been on the increase. However despite an association of testicular microlithiasis with testicular tumour [2-8] its aetiological role and the need for follow-up have remained largely unclear. The incidence of microlithiasis detection has increased with the development of more sensitive ultrasound transducers in the recent past and this has in turn increased patient anxiety in addition to the NHS workload. The cost of on going radiological surveillance of this patient group could also be phenomenal. A recent prospective follow-up study of patients with incidentally diagnosed testicular microlithiasis by Raymond A Costabile [9,10], shows no link between incidentally diagnosed testicular microlithiasis on ultrasound and testicular tumour. However the importance of testicular microlithiasis in patients with a high risk of developing testicular tumour such as cryptorchidism, small atrophic testis, sub-fertile men and testicular tumour in the contra lateral testis is not clear and needs further evaluation [12,13]. With conflicting evidence for the rationale of routinely following patients with incidentally diagnosed testicular microlithiasis [9-11] we conducted a national survey to provide a snapshot of current attitudes towards investigation and surveillance of this patient group in the United Kingdom.

## Methods

A standardised questionnaire was sent to the 464 consultants on the British Association of Urological Surgeons (BAUS) register. BAUS is the official national organisation (like the AUA in USA) of the practising urologists in the UK and Ireland. The questionnaire aimed to record individual consultants' preferred practice for managing patients in whom a finding of testicular microlithiasis is made.

Participants were asked initially whether they chose to routinely follow-up these patients and, if so, how this was achieved:

• All patients or a selected group only

• Intended duration of follow-up (life-long/up to 55 yrs of age/<5 yrs)

• Surveillance modality used (Clinical examination/ultrasound/tumour markers/Biopsy)

• Frequency of follow-up ultrasonography if utilised

Participants were also asked to record their opinion with regard to whether they felt a survival advantage was conferred for those who go on to develop a testicular malignancy by surveillance of this microlithiasis group. In addition, the participants' perceptions of the degree of association between microlithiasis and testicular carcinoma-in-situ were also requested.

## Results

Of the 464 questionnaires sent out, 263 (57%) were returned of which 12 were inadequately completed. A total of 251 were therefore analysed.

173 (69%) of the responding participants routinely choose to follow-up patients with testicular microlithiasis while 78 (31%) do not. Of those who do, 119 (69%) decide to follow-up all patients while 54 (31%) only do so for a selected patient group.

Of the 173 participants who do follow-up microlithiasis, all but one consultant used ultrasonography in addition to clinical examination as part of their surveillance. This lone consultant uses annual clinical examination as his preferred method of follow-up. While 10 (6%) of the participating consultants arrange ultrasound scan on a 6 monthly basis, a vast majority of them 151 (88%) do it annually. A further 10 (6%) scan patients at more extended intervals.

27 (16%) consultants request tumour markers in addition to ultrasound scan, while 10 (6%) would consider biopsy in selected group of patients.

With regard to the duration of follow-up these patients, 66 (38%) of the positive responders would follow-up their patients for life, while 80 (47%) would follow them up until they were 55 yrs of age. 26 (15%) would discharge their patients after 5 yrs of surveillance.

Based on their understanding of the current literature 173 (69%) participants believe testicular microlithiasis is associated with carcinoma-in-situ in less than 1% of patients. 53 (21%) felt this figure to be between 1–10% while 7 (3%) believe it to be greater than 10%.

109 (43%) participants believe that surveillance does confer a survival benefit for microlithiasis patients who go on to develop testicular malignancy while 142 (57%) do not. Interestingly, 66 (38%) responders who *do *choose to follow-up this patient group do *not *think there is a survival benefit.

## Discussion

Microlithiasis in the testis can be histological or radiological microcalification. They are not essentially the same entity. Of interest to the urologist however is the radiologically detected micolithiasis.

Oiye was the first to describe intratesticular calicifications in 6 of 192 testicles in autopsy specimens as early as1928 [14]. This report was followed a year later by Blumensaat, who reported similar intratubular bodies in postmortem specimens [15]. He felt they were degenerated spermatogonia displaced into the lumen of the seminiferous tubules. Later Bigger and Mc Adams using various histo-chemical techniques found that the laminated eosinophilic material was a glycoprotein derived from intra tubular secretions, which later calcified [16]. But it was not until 1961 that Azzopardi & Mostofi [17] from the Armed forces institute of Pathology in Washington described the two different types of intra-testicular calcification and their associated pathology. They reported the more commonly found rounded laminated intra tubular calicifications associated with cryptorchid testis, adenomatous or inflammatory pathology. They then reported the amorphous haematoxylin staining calcific bodies in dilated seminiferous tubules found in 13 of 17 patients with wide spread chorio-carcinoma. Histo-chemical methods showed them to consist of phospholipid, protein debris, DNA and calcium phosphate. These calcifications were seen in close association with malignant neoplastic cells.

Diffuse microcalcification in the testis on a plain X ray film was first reported by Priebe & Garret in a 4 year old boy with an otherwise normal testicle in 1970 [18]. But it was not until the mid 80 s when Doherty et al using a 10- MHz transducer first described ultra sonically detected testicular microlithiasis [1]. Ever since, the interest in this entity has increased, with several case reports and retrospective studies reporting an association with testicular cancer [2-8]. However these studies were either isolated case reports or retrospective studies in selected group of patients.

In one series of 263 sub-fertile men, 20% were found to have microlithiasis [12]. In the same study 20% of the men with bilateral microlithiasis were found to have CIS. Interestingly there was no association of CIS with unilateral microlithiasis in this study group. In another series of patients with testicular germ cell tumour Skakkabaek found a significant association of contra-lateral testicular microlithiasis and CIS [13]. Clearly in the high-risk group (described earlier) there seems be a significant association of testicular microlithiasis and CIS, which needs to be clarified with further longitudinal studies.

In an ultrasound screening study involving 1504 men between 18 to 35 years from the US army officer corps, Peterson & Costabile R A. [9] found the prevalence of testicular microlithiasis to be 5.6%. In this study African Americans were found to have a higher prevalence of 14% as opposed to whites who had a prevalence of 4%. However the incidence of testicular tumour is higher in whites than African Americans. Analysis of the geographical distribution of these cases showed a negative correlation with the incidence of testicular tumour in the United States. Interestingly there was an association with STD in the regions where testicular microlithiasis had a higher prevalence in this study. In their follow-up report after more than 4 years presented at the AUA meeting in 2004 at San Francisco, USA, they have not had a single case of testicular tumour in their study subjects with testicular microlithiasis.

Our survey confirms that many urologists tend to follow this patient group for a considerable period of time. However there seems to be a considerable variation in the surveillance policy. This is likely to have an enormous bearing on the cost conscious NHS practice in future. The estimated cost in the United States to follow-up all patients with microlithiasis between 18-to 35 years old are about 18-billion dollars per year [10]. It is also known from many studies that there is an average delay of 3–6 months between noticing a testicular lump and seeking medical advice without significantly affecting cure. With the cure rate for testicular cancer exceeding 90%, it is debatable whether an earlier ultrasound diagnosis will have any effect on the outcome than self examination.

With the emerging evidence it seems safe not to routinely follow-up patients who are incidentally diagnosed with testicular microilithiasis [9-11]. They should however be advised to continue testicular self-examination. The importance of testicular microlithiasis in the high-risk groups is not clear and needs further evidence. Until such time it would be logical to follow-up all patients in the high risk group.

We do acknowledge that the response rate to our questionnaire survey has been moderate, with only 57% returns. This has a small chance of bias towards urologists actively following microlithiasis returning the questionnaire, than those who are not keen on following them. However this is more likely to be due to the fact that the questionnaire was sent during school term holidays, when many urologists tend to be on annual leave. The returns were also fairly evenly distributed throughout the UK.

## Conclusion

Our survey highlights a significant variation in how patients with testicular microlithiasis are followed-up in the UK. The majority of consultants nationally believe surveillance of this patient group confers no survival benefit. However significant proportions of them continue to follow-up these patients. There is an urgent need to clarify this issue in order to recommend a coherent surveillance policy.

## Competing interests

The author(s) declare that they have no competing interests.

## Authors' contributions

SR designed the Questionnaire, conducted the survey, analysed the results and also wrote the paper. RS helped in computing and analysing the data. PAC and MVPF helped in the design of the questionnaire and overall guidance.

## Pre-publication history

The pre-publication history for this paper can be accessed here:


